# Line scan-based rapid magnetic resonance imaging of repetitive motion

**DOI:** 10.1038/s41598-021-83954-y

**Published:** 2021-02-24

**Authors:** Hankyeol Lee, Jeongtaek Lee, Jang-Yeon Park, Seung-Kyun Lee

**Affiliations:** 1 Center for Neuroscience Imaging Research, Institute for Basic Science, Suwon, South Korea; 2 Department of Biomedical Engineering, Sungkyunkwan University, Suwon, South Korea; 3 Department of Radiology, Korea University Medical Center, Seoul, South Korea; 4 Department of Intelligent Precision Healthcare Convergence, Sungkyunkwan University, Suwon, South Korea; 5 Department of Physics, Sungkyunkwan University, Suwon, South Korea

**Keywords:** Biomedical engineering, Mechanical engineering, Applied physics, Imaging techniques

## Abstract

Two-dimensional (2D) line scan-based dynamic magnetic resonance imaging (MRI) is examined as a means to capture the interior of objects under repetitive motion with high spatiotemporal resolutions. The method was demonstrated in a 9.4-T animal MRI scanner where line-by-line segmented k-space acquisition enabled recording movements of an agarose phantom and quail eggs in different conditions—raw and cooked. A custom MR-compatible actuator which utilized the Lorentz force on its wire loops in the scanner’s main magnetic field effectively induced the required periodic movements of the objects inside the magnet. The line-by-line k-space segmentation was achieved by acquiring a single k-space line for every frame in a motion period before acquisition of another line with a different phase-encode gradient in the succeeding motion period. The reconstructed time-course images accurately represented the objects’ displacements with temporal resolutions up to 5.5 ms. The proposed method can drastically increase the temporal resolution of MRI for imaging rapid periodic motion of objects while preserving adequate spatial resolution for internal details when their movements are driven by a reliable motion-inducing mechanism.

## Introduction

Magnetic resonance imaging (MRI) allows non-invasive imaging of biological tissues at the mesoscopic scale ($\backsim$ 1 mm). Although most endeavors in clinical MRI developments pertain to providing detailed images of various body parts’ static states, numerous non-clinical and clinical applications require imaging such parts in non-static conditions^1–10^. The non-static conditions may entail either physical displacements of imaged objects (e.g., in MR velocimetry and cardiac cine imaging) or secondary effects of blood flow (e.g., in functional MRI and dynamic contrast-enhanced MRI). Regardless of the non-static condition to be imaged, the need for detecting changes in MRI often mandates fast imaging capability (high temporal resolution) as well as high spatial resolution, which ensures adequate image quality necessary for observing important small-scale changes in an object. However, due to restrictions imposed by imaging principles and hardware limitations, obtaining detailed images in short scan times has been a long-standing challenge. Currently, dynamic multidimensional imaging with time scales less than 20 ms is not widely adopted in MRI, although methods to capture physical motion inside the scanner with conventional accelerated pulse sequences and longer time scales have been explored in a number of past publications^2,10–12^.

For fast, “real-time” imaging of *non-repetitive* physiological motion, variations of gradient recalled echo (GRE)-based echo planar imaging (EPI) sequences have been employed with different acceleration techniques. They can capture the movements of *in vivo* human organs with sufficient field-of-view (FOV) coverages and have been used to image e.g., laryngeal motion during speech and swallowing^11,12^.

In some cases, fast imaging is realized by limiting the imaging FOV to one-dimensional regions, as in 1D line-scan MRI^3,4^. Here, a narrow FOV is defined by saturation pulses outside the FOV, and a series of 1D line profiles of the objects’ changing states are obtained with repetition times (TR) on the order of 50 ms. This allows acquiring high temporal resolution data, albeit for a small region of interest (ROI), such as less than 10 mm for rodent functional MRI^3,4^.

Extending short-TR dynamic line scans to multidimensional imaging requires repetitive motions/changes to allow multiple k-space lines to be collected for each phase (frame) of motion. A particularly interesting and widely used dynamic imaging method for *repetitive* motion is cardiac cine imaging. Originally developed in the 1980s with spin echo pulse sequences^13,14^, this method now expansively includes various imaging techniques which can capture the cardiac motion in a video format. A key feature of cine imaging is that only a segmented section (“lines”) of the k-space of each cardiac phase is acquired per heartbeat^7,10,15^. The cine images are typically acquired with a physiological signal-triggered scan protocol, illustrating a single period of the heart cycle with high spatiotemporal resolutions. If a trigger system is unavailable or scanning without physiological signal monitoring is preferred, retrospectively triggered cine protocols can be used. In such cases, line oversampling and navigator echoes are used, where navigator data help assign each echo to its estimated position in the image retrospectively^16^. When extended scanning with many time frames are desired (e.g., short TRs), substantial amount of oversampling will be needed to reconstruct data with acceptable image quality, increasing the total acquisition time substantially^16^.

The highest temporal resolution (frame rate) in cine imaging is realized when scanning one line of the k-space in each heartbeat. However, this is often not feasible to implement because it requires many heartbeats and thereby a prolonged scan time to complete the images, which leads to artifacts due to inevitable cardiac motion irregularities and breathing. As a result, most cardiac cine imaging today utilizes segmented approach of scanning multiple k-space lines per heartbeat, at the cost of the frame rate (on the order of 20 frames per second)^17^. This compromise highlights a key component of achieving high temporal resolution in multidimensional line-scanning: reliably induced repetitive changes.

The purpose of this study is to utilize the existing 2D line-scan technique to capture artificially induced and triggered physical motion of objects inside the MRI magnet bore with very high temporal resolutions. When scanned objects exhibit no spontaneous motion while driven periodically by an external MR-compatible actuator (i.e., passively responding to the drive), line-by-line acquisition in interleaved frames can produce images with high spatiotemporal resolutions with minimal artifacts caused by motion. This practice helps achieve an uncommonly high frame rate (e.g., greater than 100 Hz), probing into the objects’ dynamic states while in motion. The study also explores a safe and reliable MR actuator design which can induce repeatable motion inside the bore. The actuator, which utilizes the Lorentz force on the wire loops in the scanner’s main magnetic field, can be triggered by the scanner to induce periodic and repetitive mechanical motion of the scanned object synchronized with the imaging pulse sequence. Our method of combining 2D line-scanning with the proposed actuator design can be used to image dynamic changes of mechanically driven rigid or soft materials.

## Methods

A 9.4-T, 30-cm-bore animal MRI scanner (Bruker Biospin, Ettlingen, Germany) and a 112/86 mm (outer/inner diameter) transmit-receive volume radio-frequency (RF) coil (Model No. T12054V3) supplied by the same manufacturer were used for data acquisition. A 2D line-scan imaging sequence modified from a vendor-provided spoiled GRE sequence was installed in the scanner and was used for all experiments described in this paper. Following the acquisition, the k-space data were processed by a custom MATLAB (MathWorks, Natick, MA) script using fast Fourier transform (FFT). This reconstruction process produced a series of temporally resolved 2D images of objects in rapid repetitive motion.

### 2D FLASH line-scanning

**Figure 1:**
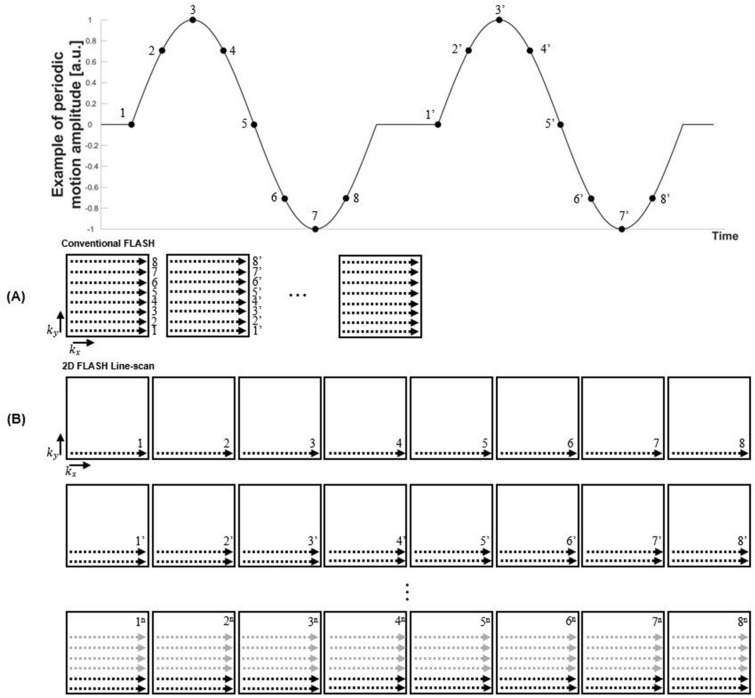
K-space filling scheme of a conventional spoiled gradient echo sequence (**A**) compared to the two-dimensional FLASH line-scan sequence (**B**).

A fast spoiled gradient echo sequence (vendor name: FLASH, fast low-angle shot) was modified for 2D line-scanning^18^ to image objects in motion with high spatiotemporal resolutions, with the minimum TR of 5.5 ms. Figure 1 illustrates the line-scan sequence’s k-space acquisition scheme. Whereas conventional FLASH imaging would acquire the entire k-space for each frame^19^, line-scanning acquires one line of k-space for each frame along the readout direction during a single period of repetitive motion. Subsequent to repeating single line acquisitions for all desired frames within one motion period using identical phase-encoding and readout gradients, a shifted phase-encoding gradient is applied and line acquisitions are repeated for all frames in the following period. This process accumulates new k-space lines parallel to the previously acquired lines. The acquisition cycle is repeated until all k-space lines are filled according to the desired resolution and FOV dimensions. Hence, the total acquisition time (TA) can be expressed as

1 $$\begin{aligned} TA&= (TR)(N_{frames})(N_{phase}). \end{aligned}$$

The total number of k-space lines to be acquired and stacked in the phase-encoding direction ($N_{phase}$) equals the number of motion periods to be completed for the whole imaging experiment.

Because only a single line of k-space is acquired per frame before proceeding to the next frame, line-scan’s frame interval is shorter than the imaging time of a corresponding 2D FLASH sequence by a factor of the matrix size along the phase-encoding direction. In other words, the temporal resolution of 2D line-scan sequence is determined by the TR, which corresponds to the acquisition time of a single k-space line. Each reconstructed image from line-scanning is a result of combining discontinuously acquired individual k-space lines.

### Actuator design and operation

Inducing mechanical movements inside the bore of an MRI scanner requires a careful approach. The scanner’s strong static magnetic field can not only cause malfunction of ordinary non-MR compatible motion-inducing devices, but also can jeopardize the safety of scanner operators when ferromagnetic elements are brought near the scanner. Previously introduced methods for inducing motion inside the bore of clinical MRI scanners range from manually perturbing a fluid sample to using small ferromagnetic materials (e.g., steel balls) for powering multiple gears connected to robotic arms^1,2,20,21^. Although such applications may be suitable for specific purposes like fluid velocimetry and surgical intervention, they cannot be easily implemented in scanners with smaller bores such as animal MRI scanners. Additionally, using a non-MR compatible material can introduce serious image quality degradation. Another potential approach to externally inducing motion is to use a pneumatic air pump that can be connected to tubes extending to the bore of the scanner^9^. This method is considered safe, but the precision of movements it can induce on scanned objects is limited.

**Figure 2:**
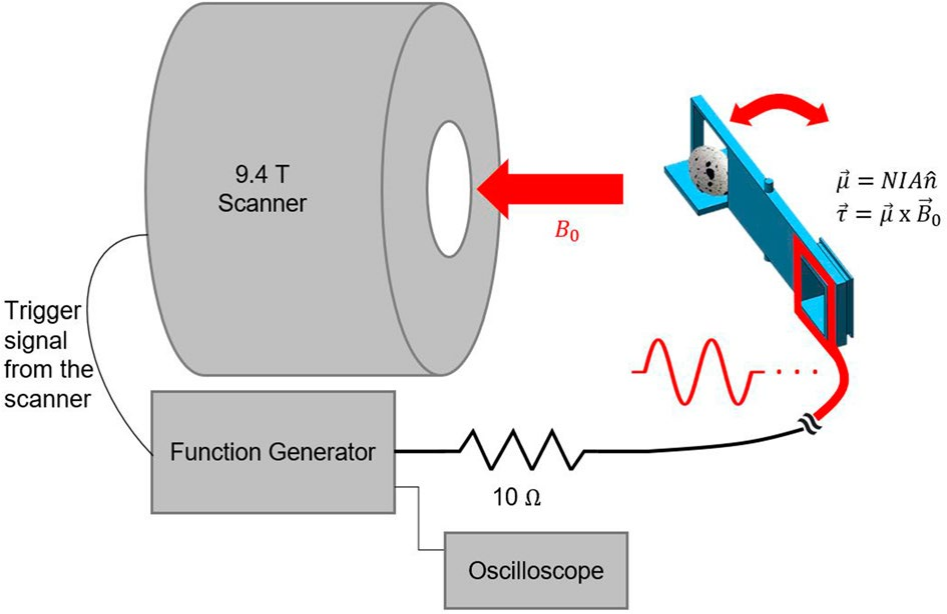
Experimental setup for imaging a quail egg in motion. The egg is attached to the 3D printed actuator arm which pivots with frequency defined by the function generator. Mechanical torque (${\vec \tau }$) on the arm is generated as the cross product of the copper wire windings’ magnetic moment (${\vec \mu }$) and the main magnetic field of the scanner (${\vec {B_0}}$).

**Figure 3:**
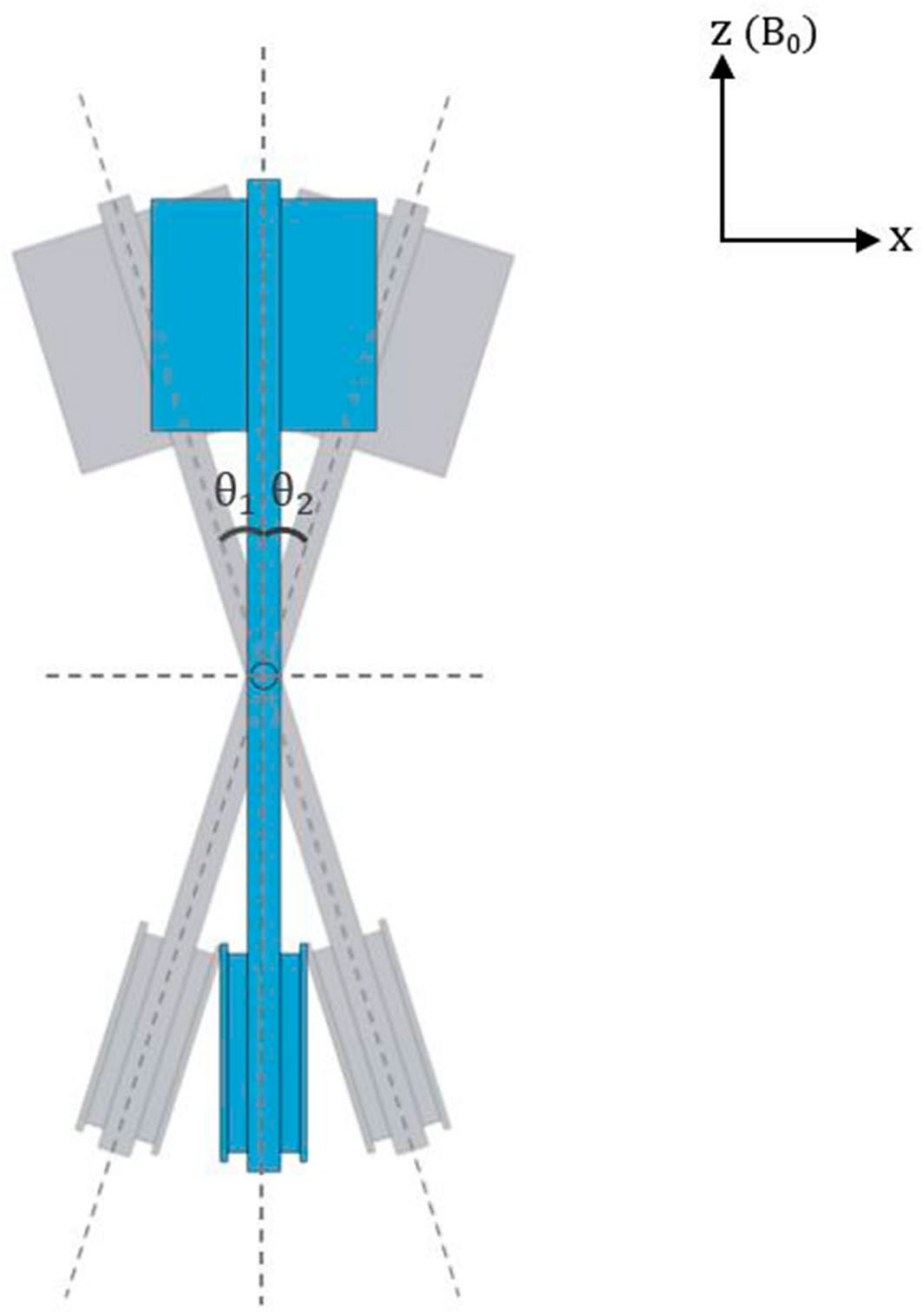
Top view of the actuator inside the bore. The actuator pivots back and forth, with maximum yaw angles in each direction (limited by the inner walls of the cradle fitted to the bore size), $\theta _1 = \theta _2 = 17.5^{\circ }$.

**Figure 4:**
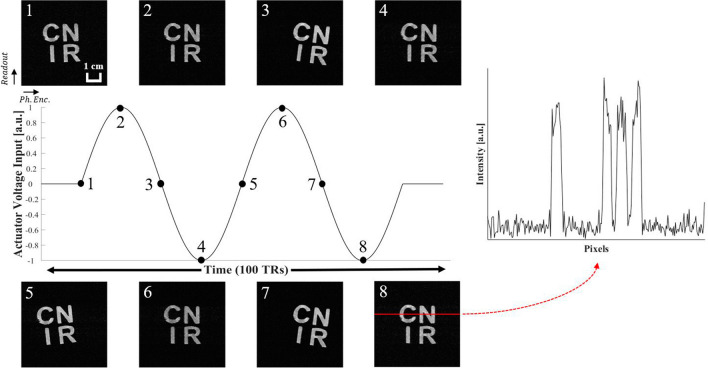
Scanning the letter-shaped agarose phantom with line-scan sequence. Two periods of sinusoidal waveforms in between a pair of brief zero intervals constituted one cycle of the actuator’s driving voltage. This was repeatedly applied to the actuator $256 (= {N_{phase}}$) times to produce the above images. The numbering on top left corner of each image indicates corresponding time points in the voltage input diagram with 10 TR-increments (800 ms). An example of linear intensity profile (red horizontal line) of Image 8 shows signal from the agarose gel clearly distinguishable from the background noise during the phantom’s movement.

In our experiments, we constructed a custom MR actuator which (i) does not contain any magnetic components, (ii) can be easily placed inside the bore of an animal scanner, and (iii) can readily interface with the scanner electrically for synchronization with the imaging sequence. The device is powered by an arbitrary waveform generator. On the scanner interface, custom triggering setup is configured to produce a square pulse during the acquisition of specifically designated image frames. The waveform generator uses the square pulses as external trigger, delivering a predefined voltage output within one motion period. This is repeated until the acquisition is over. This mechanism helps induce the desired repetitive motion of the objects inside the scanner. The actuator utilizes time-varying magnetic moments of electrical current-carrying coils, which interact with the static magnetic field of the scanner to mechanically move the objects back and forth within short periods of time.

As seen in Figs. 2 and 3, the actuator consists of a 15 cm-long 3D-printed plastic plank-like structure. An enlarged horizontal flat surface is on one end of the structure where an object to be scanned is attached. On the other end of the structure, 3.5 cm ${\times }$ 3.5 cm square edges are wrapped around by a copper wire (diameter: 0.2 mm) 23 times. The protruded cylindrical hinges located on top and bottom of the plastic structure’s center were fitted to a 3D-printed cradle with a lid. The aforementioned wire was connected to BNC cables which were extended to the control room. This cable extension was then connected to a 10 ${\Omega }$ resistor in series and a function generator which provided voltage amplitudes up to ${\pm }5$ V in sinusoidal waveforms. The net resistance of the BNC cables, the series resistor, and the wire windings was 12 ${\Omega }$, which added to the output impedance (50 ${\Omega }$) of the generator. The reactance in the windings was estimated to have negligible contribution to the impedance. The input voltage waveform was monitored on an oscilloscope. When electric current was flown in the wire, the resulting magnetic moment orthogonal to the wire loop and the main magnetic field of the scanner produced a torque which is the cross product of both vectors. Due to this torque, the scanned object experiences a rotational (pivoting) motion. This mechanism can be expressed as following:

2 $$\begin{aligned} \vec \mu&= NIA{\hat{n}} \end{aligned}$$

3 $$\begin{aligned} \vec \tau&= \vec \mu \times \vec {B_{0}} \end{aligned}$$

where ${\vec \mu }$ is the magnetic moment, *N* is the number of turns in the wire loop, *I* is the current, *A* is the area of the wire loop, ${{\hat{n}}}$ is the unit vector perpendicular to the plane of the loop, ${\vec \tau }$ is the torque, and ${\vec {B_0}}$ is the static magnetic field of the scanner. Depending on the polarity of the electric current applied to the wire, the actuator is driven in either clockwise or counter-clockwise direction. The magnitude of torque exerted by the actuator was up to 0.02 Nm with ${\pm }$ 5V generator output.

We used a driving waveform which consisted of two cycles of a sine wave sandwiched between a pair of brief intervals of zero voltages. The amplitude and frequency of the sinusoids were adjusted to achieve sufficient displacement of the imaged objects, which depended on the objects’ mass. The maximum angular displacement of the actuator was 17.5 degrees from the center limited by the walls of the cradle, which were approximately 7 cm apart from each other.

### Phantom imaging

In order to verify the performance of the 2D line scan sequence and the scanner-synchronized operation of the actuator, an experiment was conducted to image relatively slow repetitive motion (0.125 Hz) of an agarose gel phantom at a high spatial resolution. For this experiment, a 3 cm ${\times }$ 3 cm ${\times }$ 2 cm (width ${\times }$ length ${\times }$ height) plastic cube phantom was constructed using a 3D printer. The cube was printed with vertical fused deposition, representing four English alphabets (C-N-I-R) when viewed from the top. The thickness of each letter stroke varied between 2 mm and 3 mm. Prior to scanning, the letter-shaped cavities were filled with $1{\%}$ agarose solution and was attached to the actuator’s flat surface using a strip of surgical tape. The phantom weighed 20 g. In order to acquire images showing distinguishable letters, a high spatial resolution was desired. The scan parameters were: $\hbox {TR/TE} = 80/2.504\,\hbox {ms}$, flip $\hbox {angle} = 30{^\circ }$, $\hbox {bandwidth} = 391\,\hbox {Hz/pixel}$, $\hbox {slice-thickness} = 1\,\hbox {mm}$, matrix $\hbox {size} = 256 \times$ 256, FOV = 58 $\times$ 58 $\hbox {mm}{^{2}}$, and pixel $\hbox {size} = 0.23 \times 0.23$ $\hbox {mm}{^{2}}$. The total acquisition time was: $\hbox {TA} = 80\,\hbox {ms}$ (TR) $\times$ 100 ${(N_{frame})}$ $\times$ 256 ${(N_{phase})} = 34$ min 8 sec.

### Quail egg imaging

The 2D line-scan’s capability of capturing rapid fluid motions was demonstrated by imaging periodically driven (1.82 Hz) quail eggs in two conditions: (i) raw (liquid contained by shell) and (ii) fully cooked and deshelled (solid). Like the agarose phantom, the eggs (approximately 10g each) were fixed in position on the flat surface of the actuator rotor with tape. Compared to the gel phantom imaging, high temporal resolution was given priority over spatial resolution in order to image rapid fluid disturbances occurring inside the raw egg. As a result, the following imaging parameters were used: TR/TE = 5.5/2.502 ms, flip $\hbox {angle} = 9{^\circ }$, bandwidth = 391 Hz/pixel, slice-thickness = 2 mm, matrix size = 128 $\times$ 128, FOV = 55 $\times$ 55 $\hbox {mm}{^{2}}$, and pixel size = 0.43 $\times$ 0.43 $\hbox {mm}{^{2}}$. The total acquisition time was: TA = 5.5 ms (TR) $\times$ 100 ${(N_{frame})}$ $\times$ 128 ${(N_{phase})}$ = 1 min 10 s.

## Results

**Figure 5:**
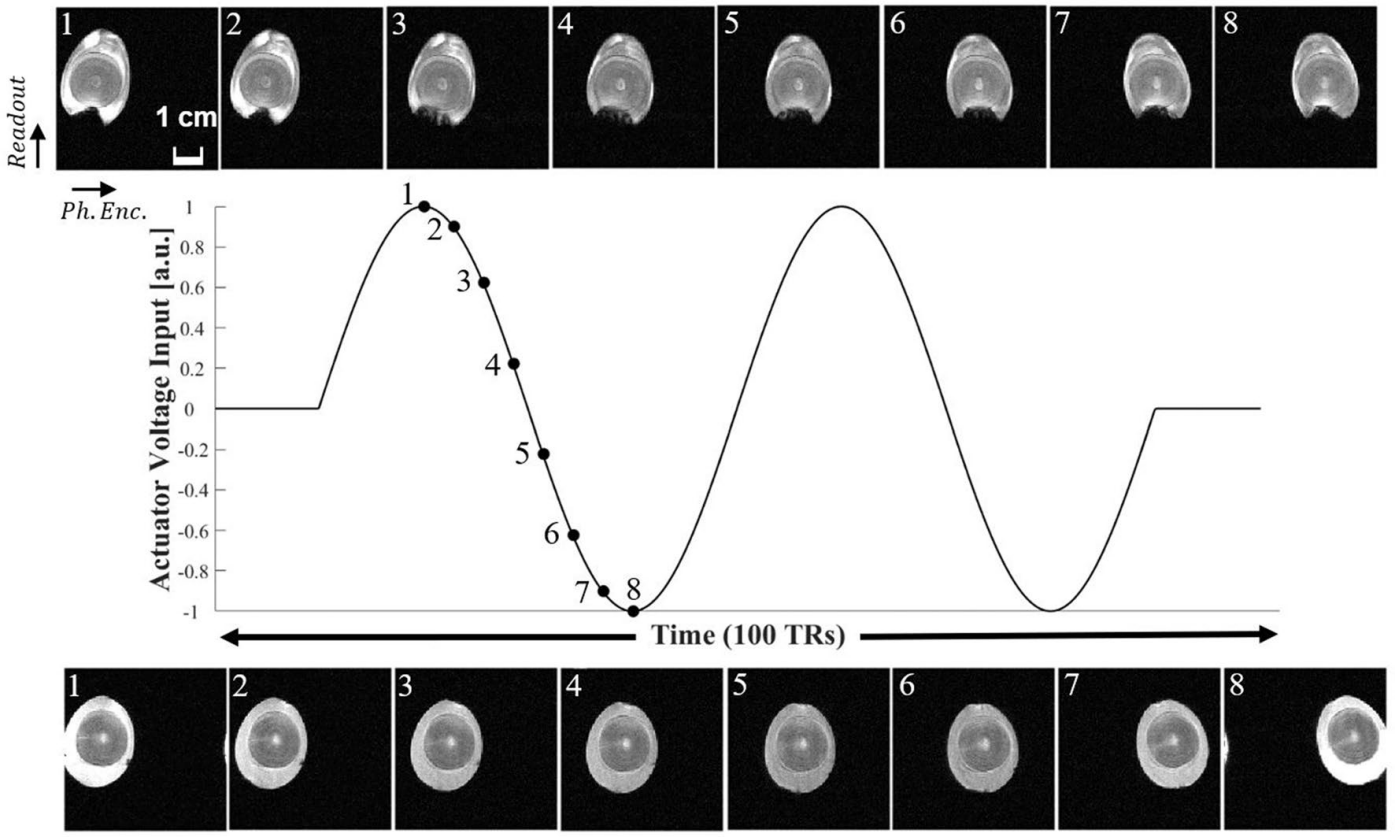
Time-course images of raw and cooked quail eggs in motion, displayed on top and bottom of the figure, respectively. The driving voltage contained two periods (220 ms each) of a sinusoidal wave. Images of raw egg containing viscous fluid show the egg’s air cell producing patterns of fluid motion. On the other hand, cooked and de-shelled egg shows no sign of fluid motion. Numbering on top left corner of each image indicates relative time points in the voltage input diagram with 3 TR-increments (16.5 ms).

2D FLASH line-scan’s discontinuous k-space acquisition method made capturing of the fast and repetitive movements possible. Figures  4 and 5 display images representing the objects (phantom and eggs) at different time points in the actuator’s motion cycle, which consisted of 100 TRs.

Figure 4 shows time-course images of the agarose phantom during its periodic movements. The eight time-course images displayed were acquired 10 TRs apart from each other. During the acquisition, two periods of sinusoidal wave voltage (3.2 s period), with prepending and appending zeros, were applied to the actuator. The total voltage waveform was 100 TRs (= 8000 ms) long, and was repeated 256 times, which equals the number of the lines along the phase encoding direction. In the reconstructed time-course images, all letters in the phantom can be clearly identified during its movement. This can be reconfirmed in the linear intensity plot in Fig. 4. The pixels occupied by the agarose gel had a well-defined intensity profile with little smoothing or blurring effect from the nearby empty pixels even during the phantom’s movement.

In Fig. 5, time-course images of quail eggs in motion are shown. The example images displayed were acquired with 3 TR increments for both raw and cooked eggs. As in the phantom imaging, a 100-TR-long waveform containing double sinusoids was repeated. The period of each sinusoid was 220 ms. In the raw egg, the signal void at the bottom was caused by an inner air pocket and its fluid motion was captured in the time-course images. However, such fluid motion was not observed in the de-shelled solid (cooked) egg.

**Figure 6:**
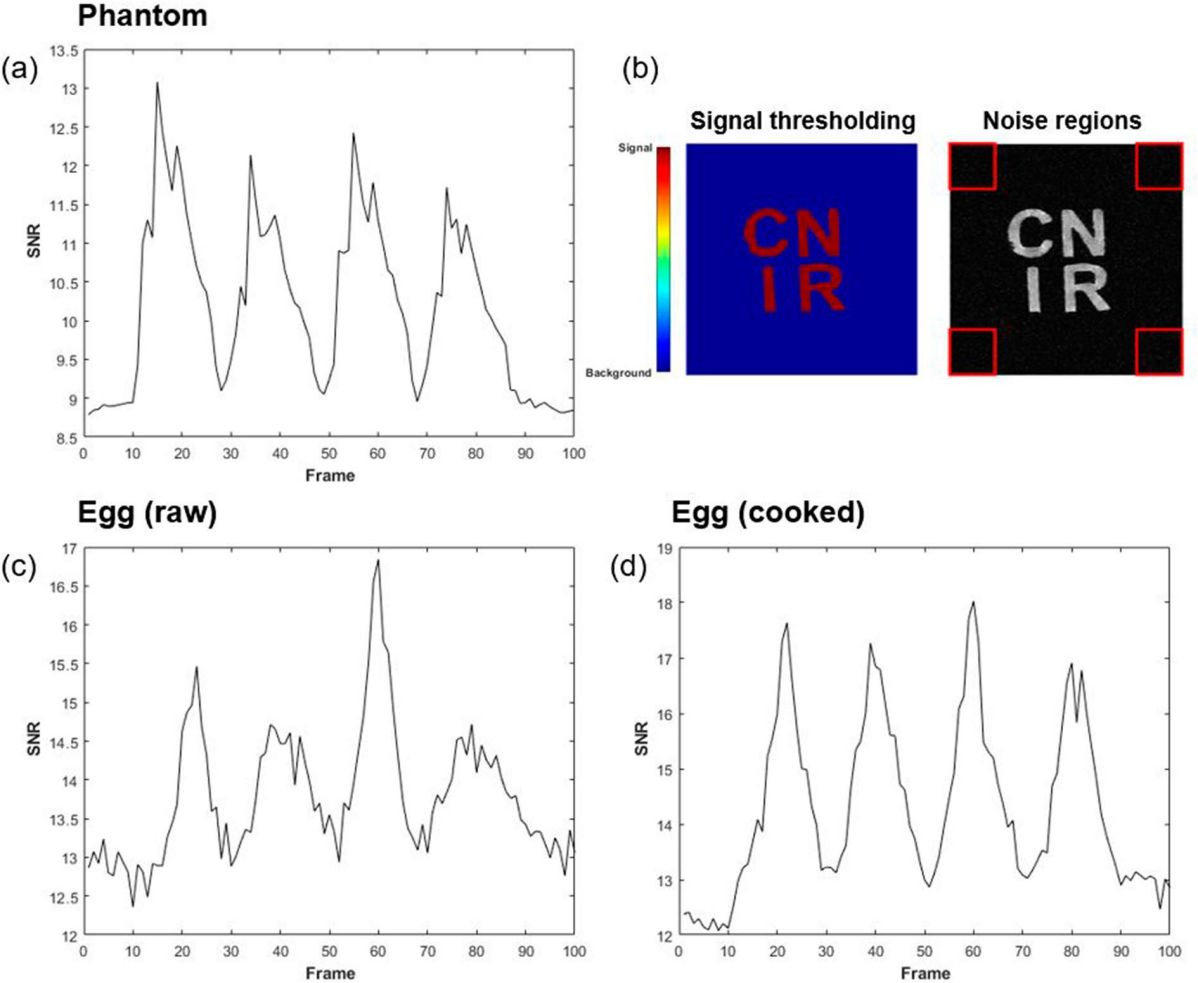
Time-course SNR analysis of agarose phantom (**a**) and raw/cooked eggs (**c**,**d**) in motion, during one period of driving voltage corresponding to the double-sinusoidal motions shown in Figs. 4 and 5. The SNR increased when both objects approached the RF coil elements when swung to the maximum displacement position by the actuator. In all cases, signals from scanned objects were isolated using intensity thresholding while noise regions were defined as four corners of each image (**b**).

The signal-to-noise ratios (SNRs) of all objects varied during their motion. Figure 6 illustrates time-course SNR measurements for all scanned objects. SNR was defined as the ratio of the mean signal (taken over the entire object using intensity thresholds) to the root mean square of noise defined in four square ROIs in each corner of all images, where the ROI matrices varied between 50 ${\times }$ 50 for the phantom, and 20 ${\times }$ 20 for the eggs. During the objects’ movements toward and away from the walls of the cradle, periodic variations of the SNR were observed for all scanned objects. When the objects were closer to the wall, the SNR increased by up to $49{\%}$ (phantom), $41{\%}$ (raw egg), and $54{\%}$ (cooked egg) compared to when they were at the center. This is most likely due to the increased signal sensitivity near the coil elements when the actuator arm holding the object reached the furthest in each direction.

**Figure 7:**
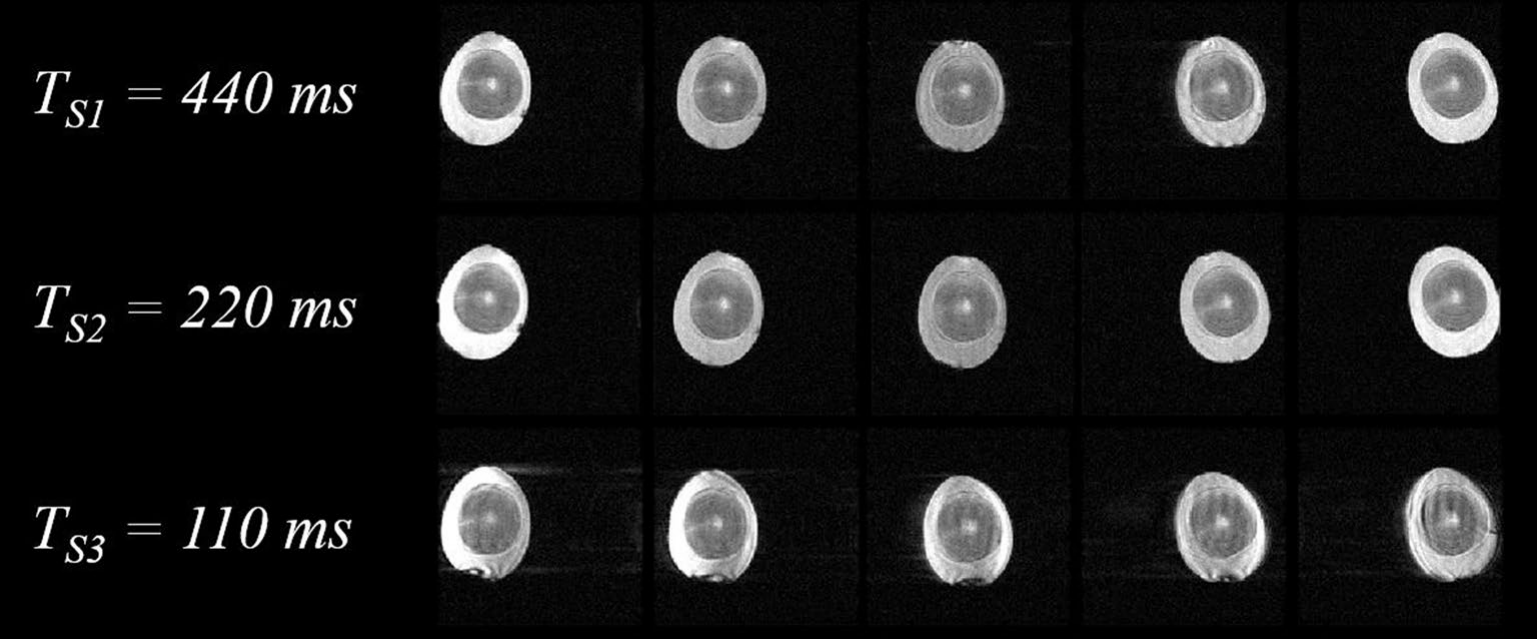
Images of cooked egg with different rotational motion speeds in their relative positions. The sinusoidal periods ($T_{S}$) of voltage input to the actuator (and thus the swing motion period) were 440 ms, 220 ms, and 110 ms for $T_{S1}$, $T_{S2}$, and $T_{S3}$, respectively. Increased level of image artifact is observed for the fastest motion at $T_{S3}$.

Even with the increased motion frequency applied to the eggs (compared to the slower movement of the phantom), the reconstructed images maintained good SNR and continued to display the interior of the eggs clearly at all positions. However, when the egg was swung with faster motion (${\times }2$ compared to Fig. 5), with a 110 ms-period sinusoidal drive, motion-related artifacts became more noticeable, as shown in Fig. 7.

## Discussion

Our implementation of 2D FLASH-based line-scanning has a capability of imaging objects with a high temporal resolution compared to other conventional scan protocols such as EPI and GRE sequences. It should be noted, however, that an important restriction exists. Whereas conventional pulse sequences most often acquire an entire k-space within a continuous time period (i.e. slice or volume TR), 2D line-scan only acquires a single line of the k-space for a given motion period. This k-space is then left incompletely filled before another line of the same k-space is acquired in the next period. Such discontinuity in the acquisition of the k-space restricts the application of the 2D line-scan sequence to highly repeatable and cyclical motions, which ideally need to be artificially manipulated. When such motion is synchronized with the imaging sequence correctly, 2D line-scan can effectively capture rapid repetitive movements.

It is worth mentioning that further increase of temporal resolution may be achieved by encoding each frame of motion with phase-encoding gradients only, which can last for less than a millisecond. This has been demonstrated to image periodic oscillation in one dimension^22^. However, extension of this method to multidimensional movements will take excessive scan times.

The 2D line-scan technique presented in this paper was based on a simple GRE sequence and limited to single-slice imaging to achieve a high temporal resolution (5.5 ms) while having a reasonable scan time. Further adaptation of the multidimensional line-scanning method to other pulse sequences will be straightforward to implement. For example, three-dimensional (3D) extension of the line-scan, through addition of phase encoding steps in the third dimension, can be useful for more comprehensive dynamic imaging. More sophisticated coil setup with a multi-receiver array can enable parallel imaging, including simultaneous multislice (SMS) imaging, for scan acceleration^23–27^. Such adaptation may be essential to keep the total scan time reasonably short even with 3D imaging.

Higher-dimensional sequence implementation can also be combined with more sophisticated, remotely controlled actuator devices inside the scanner bore. Although a simple actuator design connected to a triggered power source (function generator) was demonstrated here as a proof-of-concept, attaching mechanical gears to the actuator or multiple wire loops driven by independent power sources can also be used for moving objects in multiple directions inside the scanner, including 360-degree full rotations. If coupled with the multidimensional line-scan technique for dynamic imaging with high temporal resolution, the mechanism can introduce the possibility of scanning objects in a variety of driven repetitive motion to probe their mechanical responses.

Our work presents the first step towards utilizing MRI for direct visualization of the dynamic response of biologically relevant materials to mechanical actuation. While clinical application is currently difficult due to the requirement of precise and extended periodic motion, the proposed method can be highly relevant in the investigation of mechanical properties of *ex vivo* or engineered biological organs^28,29^. In order to further exploit the versatility of MRI in soft tissue imaging, the proposed multidimensional line-scan methodology can also be combined with different signal read-out schemes to highlight different tissue contrasts and optimize the SNR. For example, to image short-$\hbox {T2}{^*}$ materials in motion, ultra-short echo time (UTE) line-scanning in combination with non-Cartesian (spiral or radial) k-space trajectories can be considered^30,31^. On the other hand, balanced steady-state free precession (bSSFP) line-scan may help achieve greater SNR per scan time despite the increased sensitivity to $\hbox {B}{_0}$ inhomogeneity^32^. As our dynamic imaging involves gross motion of the imaged objects, position- and orientation-dependent $\hbox {B}{_0}$ and RF field inhomogeneity would need to be carefully considered and compensated^33^ in order to avoid spurious dynamic contrast variation. Additionally, while our method provides much higher temporal resolution than conventional imaging, motion artifacts will still appear at highly accelerated movements as observed in Fig. 7. More systematic investigation of the artifact mechanisms of the line-scan method, including motion artifacts^34^, remains as a subject of future research.

## Conclusion

In this article, a 2D slice-imaging line-scan technique was investigated along with a custom MR actuator as a means to capture rapid repetitive movements. This capability was demonstrated by scanning an agarose phantom and quail eggs in different conditions. The reconstructed images effectively displayed the time-course displacements of the objects with minimal movement artifacts. When coupled with repetitive modulation of the movements, the line-by-line k-space acquisition scheme can enable detection of the mechanical response of various scanned objects with very high spatiotemporal resolutions.

## Supplementary Information

Supplementary Information 1.

Supplementary Information 2.

Supplementary Information 3.
