# Supplementary material for: Line scan-based rapid magnetic resonance imaging of repetitive motion
Source: Sci Rep. 2021 Feb 24;11:4505. doi: 10.1038/s41598-021-83954-y (PMC7904786; doi:10.1038/s41598-021-83954-y)
Supplement: Supplementary file 1 — Supplementary Information 1. [file 41598_2021_83954_MOESM1_ESM.pdf]

Supplementary Information

Line scan-based rapid magnetic resonance imaging of repetitive motion

Hankyeol Lee[1], Jeongtaek Lee[2,3], Jang-Yeon Park[2,4], and Seung-Kyun Lee[1,2,4,5,*]

[1]Center for Neuroscience Imaging Research, Institute for Basic Science, Suwon, South Korea
[2]Department of Biomedical Engineering, Sungkyunkwan University, Suwon, South Korea
[3]Department of Radiology, Korea University Medical Center, Seoul, South Korea
[4]Department of Intelligent Precision Healthcare Convergence, Sungkyunkwan University, Suwon, South Korea
[5]Department of Physics, Sungkyunkwan University, Suwon, South Korea
[*]Corresponding author, seungkyun@skku.edu

Supplementary Video 1. Pivoting raw egg motion recorded with 2D line-scan protocol (TR = 5.5 ms). The motion was driven by sinusoidal voltage input to the acutator (220 ms period per sinusoid).

Supplementary Video 2. Pivoting cooked egg motion recorded with 2D line-scan protocol (TR = 5.5 ms). The motion was driven by sinusoidal voltage input to the acutator (220 ms period per sinusoid).
